# Supplementary material for: Class III Peroxidases in the Peach (Prunus persica): Genome-Wide Identification and Functional Analysis
Source: Plants (Basel). 2024 Jan 2;13(1):127. doi: 10.3390/plants13010127 (PMC10780707; doi:10.3390/plants13010127)
Supplement: Supplementary file 1 [file plants-13-00127-s001.zip › Supplementary Table S1.pdf]

Table S1. Characteristics of peach cultivars

Cultivar characteristic	*Cultivars*					
	**"Asmic"**	**"Springold"**	**"Fantasia"**	**"Venus"**	**"Cold Princess"**	**"21th Century"**
Frost resistance	cold-tolerant (Sychov, 2018)	cold-tolerant (Moale et al., 2013, 2016)	-cultivars with a medium break of dormancy (Horsáková, Krška, 2016) -cold-tolerant (Scorza, Okie, 1991)	-cold-tolerant (Szalay et al., 1999)	cold-tolerant (Li et al., 2021)	cold-sensitive (Li et al., 2021)
Drought resistance	drought-resistant (Fedorova et al., 2021; Tsiupka et al., 2022)	drought-resistant (Steinberg et al., 1988; Ivashchenko et al., 2017, Tsiupka et al., 2023)	drought-resistant	drought-resistant	-	-
Resistance to biotic stressors	*Taphrina deformans* – resistant, *Sphaerotheca pannosa* – medium resistance/ sensitiv; *Clasterosporium carpohilum* - resistant	*Taphrina deformans* – medium resistance; *Cytospora cincta* - medium resistance/ sensitiv; *Monilinia Laxa* - medium resistance/ sensitiv; *Monilinia Fructigena* - medium resistance (Moale et al., 2022)	*Taphrina deformans* – medium resistance; *Cytospora cincta* - medium resistance; *Sphaerotheca pannosa* – medium resistance (Melnichenko, 2008)	*Taphrina deformans* – resistance; *Cytospora cincta* – medium resistance; *Sphaerotheca pannosa* – medium resistance; *Clasterosporium carpohilum* – medium sensitiv	-	-
Fruit characteristics	Large-fruited variety (Sychov, 2018), medium-late ripening (end of July), flesh color - white, fruit consistency - fibrous (Smykov et al., 2015)	Medium and small fruits (Okie, 1998); Spherical, yellow with 30% red Yellow, ferm, good quality (Gavăț,1990), mid-ripening (early July), green-meaty, fibrous, the bone comes off (Smykov	Slow maturing phenotype (Farinati,2021) Round-oval, sweet and sour nectarine with detachable stone (Dirlewanger, 2006), with firm flesh (You, 2021)	Self-fertile, high-yielding mid-early nectarine cultivar. the fruits are red-yellow attractive in color, large ones reaching up to 200 grams. The pulp is yellow, slightly reddish, juicy, pleasant taste, the stone does not separate	Spherical or flattened shape, large, hard texture, red skin, but white pulp, slightly sour taste	

		et al., 2015)		from the pulp. (Carrasco-Valenzuela et al., 2019; Balsells-Llauradó et al., 2023)		
